# Humans Use Predictive Gaze Strategies to Target Waypoints for Steering

**DOI:** 10.1038/s41598-019-44723-0

**Published:** 2019-06-06

**Authors:** Samuel Tuhkanen, Jami Pekkanen, Paavo Rinkkala, Callum Mole, Richard M. Wilkie, Otto Lappi

**Affiliations:** 1 Cognitive Science, Department of Digital Humanities & Helsinki Centre for Digital Humanities (Heldig), University of Helsinki, Helsinki, Finland; 2 TRUlab, University of Helsinki, Helsinki, Finland; 3 School of Psychology, University of Leeds, Leeds, UK

**Keywords:** Saccades, Smooth pursuit, Human behaviour

## Abstract

A major unresolved question in understanding visually guided locomotion in humans is whether actions are driven solely by the immediately available optical information (model-free online control mechanisms), or whether internal models have a role in anticipating the future path. We designed two experiments to investigate this issue, measuring spontaneous gaze behaviour while steering, and predictive gaze behaviour when future path information was withheld. In Experiment 1 participants (N = 15) steered along a winding path with rich optic flow: gaze patterns were consistent with tracking waypoints on the future path 1–3 s ahead. In Experiment 2, participants (N = 12) followed a path presented only in the form of visual waypoints located on an otherwise featureless ground plane. New waypoints appeared periodically every 0.75 s and predictably 2 s ahead, except in 25% of the cases the waypoint at the expected location was not displayed. In these cases, there were always other visible waypoints for the participant to fixate, yet participants continued to make saccades to the empty, but predictable, waypoint locations (in line with internal models of the future path guiding gaze fixations). This would not be expected based upon existing model-free online steering control models, and strongly points to a need for models of steering control to include mechanisms for predictive gaze control that support anticipatory path following behaviours.

## Introduction

In humans and many other animals, the visual system allows information relevant for guiding locomotion to be sampled at some distance from the body. This enables anticipatory steering control, evidenced in smooth steering appropriate to upcoming path constraints^1–9^. The typical pattern of gaze during natural locomotion (free-gaze, head-unrestrained) can be characterized as looking where you are going, and steering where you look; in other words, gaze and steering are closely coordinated in natural visuomotor strategies^3,10–12^.

However, while the where and when of human gaze behaviour when steering has been investigated in a number of careful observational studies in highly naturalistic conditions – including field experiments in the wild using mobile eye tracking^3,10,13^ (for reviews see^14,15^) – even the most accurate naturalistic observational techniques cannot resolve the mechanisms underpinning oculomotor and locomotor coordination. Experimental manipulations in controlled conditions are needed to tease apart the predictions of different theories.

The most fundamental theoretical issue concerns whether behaviour in visually guided locomotion is controlled by *internal models*, analogous to those posited for sensorimotor control in other domains^16–20^, for sensory integration in other primates^21–23^ and even invertebrate behavior^24^. The alternative would be that locomotor behaviour is better accounted for by *model-free* online control mechanisms driven by optically available information, attuned to relevant environmental cues^25^ (for a review of model-free locomotor control see^5^). Both approaches can account for anticipatory steering but do so in very different ways. In online models anticipatory control is *prospective*, meaning that steering is controlled in response to directly available optical cues that are in themselves predictive about upcoming steering requirements^5,10^. In contrast, for internal models anticipatory control is *predictive*: current and past observations are integrated to update an internal model – an estimate of the world state which the brain uses to predict its own input, using prediction-error feedback to update the model and action choice^26–30^. The internal model can be used to support forward-prediction of future states, and action planning (see^9^ for a theoretical discussion of the origins and fundamental assumptions of the two approaches in the domain of visuomotor steering control).

So far, neither approach has produced integrative computational accounts of both the mechanisms for actively orienting gaze to the appropriate targets and the mechanisms for controlling steering on the basis of the perceptual information picked up. Model-based control could provide a framework for a set of generalizable mechanisms, allowing, for example, on-demand translations between reference frames, as well as transfer of learning by reusing similar perceptual and predictive mechanisms across tasks. There is, however, a dearth of direct evidence supporting the *need* for positing internal models in the visual locomotor control literature^25^. Given the rich pattern of potential stimulus cues present in naturalistic conditions, it is not trivial to design an experiment where performance could not plausibly be explained by purely online processes. The examination of this question effectively requires a scene where external visual cues can be removed, but at the same time allows the human to exhibit behaviours that are close to naturalistic behaviour, *and* moreover would only be expected based on an internal model.

So, although a model-based approach to perception and control would potentially integrate the visual control of steering literature with the literature of gaze control as prediction^31^ and the ‘predictive brain’ framework^30^, there is at present little experimental work that would provide direct quantitative support for the development of such predictive-processing models.

This paper reports two experiments that bridge this gap. In our first experiment (See Movies 1, 2 and 3 for sample trials, link to the movies: https://zenodo.org/record/2592395) we use eye tracking in a simple steering task to assess spontaneous fixation placement in displays with rich optic flow, and in an extended behavioural sequence (as opposed to short trials of a few seconds’ duration only). The results of Experiment 1 show that the participants systematically use a gaze strategy of *fixating waypoints* on their future path^5,9,32,33^. Reliably establishing where participants naturally look gave us the necessary parametric understanding to create, for the second experiment, a matched path that was only specified by a series of such waypoints. Specifically, in Experiment 2 we removed the majority of optic flow and path information – but retained a minimum stimulus configuration sufficient to elicit natural gaze behaviour (see Movie 4 for a sample trial). This allowed us to probe whether a waypoint needs to be a steering point *specified by optical features* in order to shift gaze to the next waypoint, or whether *predictive waypoint fixation of unseen waypoints* would be elicited by intermittently withholding the relevant visual information. The waypoints were placed predictably at regular intervals, but 25% of the waypoints were masked to determine whether participants still looked toward the predicted location, or kept gaze directed towards visible waypoints. We observed reliable anticipatory saccades towards the (invisible) waypoint location, a behaviour that would not be predicted by online, stimulus-driven accounts of fixation placement. Combined, the results from these two experiments point to a need for visuomotor steering models to include mechanisms for predictive gaze strategies.

## Results

### Quantifying natural gaze fixations when steering a winding path

**Figure 1:**
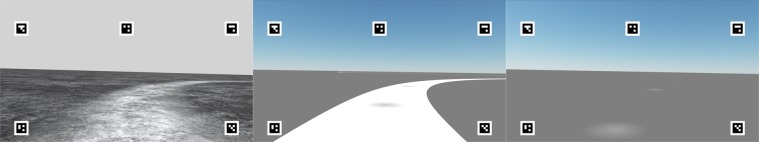
Stimuli. *Left panel*. Example display from Experiment 1 (Movies 1–3 shows gaze location marked by a red dot). The rich texture was designed to give realistic-looking visual flow (see Experiment 1 Stimuli and Design in the Methods for details), and extensively piloted to reduce aliasing at the natural fixation distance (which can create a steady-state appearance visual artefact). *Centre panel*. Example display of a practice trial from Experiment 2 with waypoints superimposed at regular intervals onto the same shaped path as used in Experiment 1. Road and waypoints were both visible. Note that the ground and path are untextured; the only self-motion cues for steering are the edge lines and waypoint motion, waypoint expansion, and simulated vehicle roll. *Right panel*. Experiment 2 test trials removed the white path so only waypoints were visible on the otherwise featureless ground (see Movie 4): the path and self-motion are designated only by the waypoints’ motion and visual expansion (and vehicle roll). In all displays the square optical markers around the edges were used during image processing to determine a homography from the eye tracker’s camera to the screen.

The first experiment recorded natural gaze fixation behaviours of humans steering along a winding path (Fig. 1, left panel; Movie 1; see Methods for details). Fifteen participants drove on a simulated track consisting of successive 50 m radius half-circles in alternating directions for 1 minute. Three speeds (40 km/h, 53 km/h, and 66 km/h, yielding yaw rates of approximately 13 deg/s, 17 deg/s and 21 deg/s) were used in an ascending sequence. The participants sat in a gaming seat and controlled the virtual vehicle with a steering wheel. Road and ground textures were designed with the goal of generating realistic-looking visual flow at the typical fixation distance. To avoid presenting spurious gaze targets, a minimal layout was adopted with no semantic cues such as trees, rocks, or salient road edges (Fig. 1). The horizontal field of view of the screen on which the virtual scene was displayed was approximately 70 degrees when viewed from 0.85 m. No gaze or driving line instructions were given other than to drive along the track and stay on the path.

In real-world conditions, humans spontaneously exhibit complex ‘active gaze’ patterns when steering such paths^9^. Most commonly guiding fixations (GFs) – preceding actions by about 1 s lead time, and directed to the ground about 1–2 s ahead of the current location – are interleaved with occasional look-ahead fixations much further ahead. Such *gaze polling* behaviour has been observed both in the lab^33,34^, and in a number of on-road studies^13,35–37^. Similar gaze behaviour has also been observed while walking in natural terrain^12^.

Because GFs reliably precede steering actions they are commonly assumed to be crucial for gleaning preview information about imminent needs to change speed or heading^3,14,33,37–39^ (for a review of the GF/steering literature see^8^). In our analysis, we focused on the GF parameters: where in the scene the preview information for guidance was sampled, and the oculomotor characteristics of this sampling process (specifically, whether gaze would fixate and track waypoints, or travel along the path smoothly). In particular, we wanted to examine the time headway (TH) of gaze – the time it would take for the driver to travel to where gaze was directed. We reasoned this would also serve to validate the placement and presentation schedule of the waypoints in Experiment 2.

**Figure 2:**
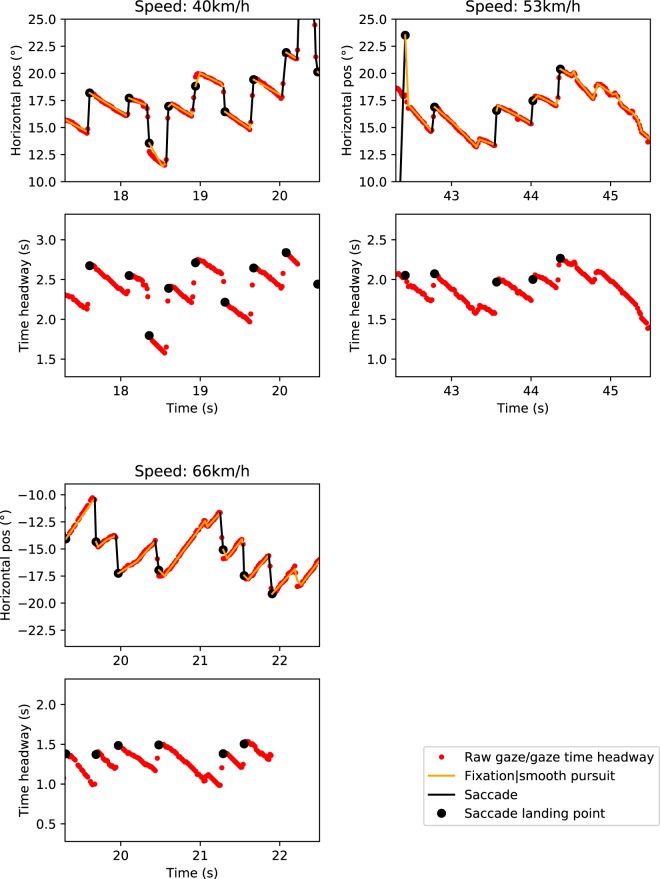
Gaze behavior and saccade identification. Sample time series of horizontal gaze position and gaze time headways in all the speed conditions of Experiment 1 (participant 8 in Supplementary Table 1). Note that with waypoint-tracking OKN the time headway of a fixation is not constant as it would be when looking at a travel point.

The data clearly show that the participants produce eye movement patterns with optokinetic nystagmus (OKN) properties: alternating between slow phase eye movements and saccadic eye movements (Fig. 2, see also Movies 1–3 for sample videos of the three speed conditions). This pattern is consistent with the participants using optokinetic reflex and pursuit mechanisms to track *waypoints* positioned on the future path (cf.^5,9,11,12,32,33,40,41^, although, crucially, no discrete visible waypoints distinct from the surrounding texture are visually specified in the flow field (nor were there any gaze instructions). Note that we’re not positing one way or another the degree to which these eye movements were voluntary or reflexive, only that the eye movement patterns are consistent with pursuing features that are moving in the visual field (and toward the driver during the slow-phases in this case). This behaviour would not be predicted by steering models that posit guiding fixations to travel points moving with the observer^6,10^ (for the distinction between travel points and waypoints, and a review of the models see^8^).

In order to give a range estimate of the time headway of guiding ‘fixations’ (fixation here refers to fixing one’s gaze to an object or target location which may well move), the closest point on the trajectory of the driver from both the landing point of each identified saccade, i.e. beginning of a new pursuit/fixation, and the launch point of the following saccade, i.e. the end of the pursuit/fixation, was identified. The trajectory of the driver was determined from the telemetry of the virtual vehicle. The time headway of the saccade landing and launch points was determined as the length of time it took for the participant to travel to the point of fixation (from where they were when the saccade landed/was launched). If a gaze point was more than 3 degrees away from the driver’s trajectory, no time headway was calculated. As an additional measure, we determined the duration between successive saccade launch points and landing points to estimate how long the pursuits and fixations were.

**Figure 3:**
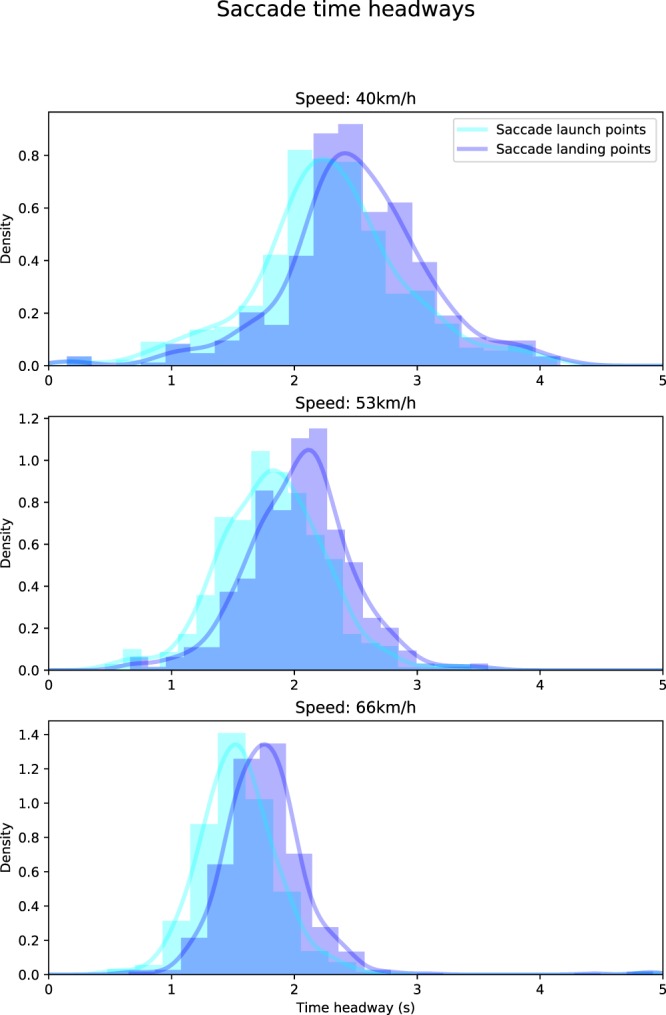
Saccade launch and landing point distributions. Gaussian kernel density estimates and histograms of the time headways of saccade launch points (cyan) and landing points (purple; black dots in Fig. 2). The data were pooled across all participants. The panels show data for the three different speeds in Experiment 1. Both the time headways and the amount of variation appear to drop as the speed increases (see Table 1 for between-subjects deviations and Table 2 for within-subjects deviations. See Supplementary Fig. S1 for analysis of the increase in speed and the distance of saccade landing points).

Each of the saccade measures (launch point TH, landing point TH, and duration between saccades) was estimated separately for each speed condition (see Table 1 and Fig. 3; Supplementary Table 1 shows the medians of each individual participant). The fixation duration (OKN slow phase duration) was around 0.4 s in every speed condition. Interestingly, the subject median saccade landing point time headways consistently decreased as the speed increased (Pearson’s r = −0.83), as did the saccade launch point time headways (Pearson’s r = −0.80). The drivers’ gaze does move farther, but this behaviour does not fully compensate for the fact that with a higher speed the equivalent time headway is also much farther ahead (see the Supplementary Analyses and Results for more detailed comments on this). At the same time, the increase in speed reduces the variability in the point of fixation time headways (see Table 2); correlation of within-subject standard deviation with speed is significant for both saccade landing point THs (Pearsons’ r = −0.3, p = 0.05) and launch point THs (Pearson’s r = −0.43, p = 0.003).

**Table 1:** Mean (of participant median) saccade launch point time headways (THs), landing point time headways, and duration between saccade landing and launch point in each speed condition in Experiment 1, as well as the respective standard deviations of the subject medians.

Speed condition	Saccade launch point THs (mean, seconds)	Saccade landing point THs (mean, seconds)	Interval between saccade landing and launch points (mean, seconds)
40 km/h	2.36, SD = 0.32	2.61, SD = 0.30	0.38, SD = 0.10
53 km/h	1.87, SD = 0.23	2.10, SD = 0.23	0.37, SD = 0.08
66 km/h	1.55, SD = 0.16	1.76, SD = 0.14	0.38, SD = 0.11

**Table 2:** Median within-subject standard deviations of the saccade landing and launch point time headways in each speed condition in Experiment 1, as well as the respective between-subjects standard deviations of the within-subject deviations.

Speed condition	Saccade launch point TH SDs (within-subject medians, seconds)	Saccade landing point TH SDs (within-subject medians, seconds)
40 km/h	0.46, SD = 0.21	0.40, SD = 0.17
53 km/h	0.31, SD = 0.12	0.28, SD = 0.12
66 km/h	0.23, SD = 0.19	0.23, SD = 0.20

The time headways of saccade landing points were consistent with the values reported for GF in the literature (around 1–3 s for both the launch and landing points). This is in line with previous observations in both laboratory conditions^7^ and in real-world driving^37^.

### Quantifying natural gaze fixations when steering via a series of waypoints

In Experiment 2, the rich visual flow and texture-delineated path were replaced by presenting the participants *only* intermittently appearing waypoints on a featureless ground plane. Twelve human participants drove along a track consisting of interconnected semicircular curves alternating left and right as in Experiment 1, but the ground texture was replaced with a solid grey colour. The track was delineated by waypoints rendered as 0.7 m radius circles on the ground using a grey-white texture that faded linearly to blend with the grey ground colour toward the edges (see Fig. 1, right panel). The speed of the vehicle was automatically kept at approximately 47 km/h. Each waypoint became visible with a 2 second time headway (i.e. 2 s before arriving at the waypoint), which was appropriate for participants to target them with guiding fixations according to literature and results from Experiment 1.

**Figure 4:**
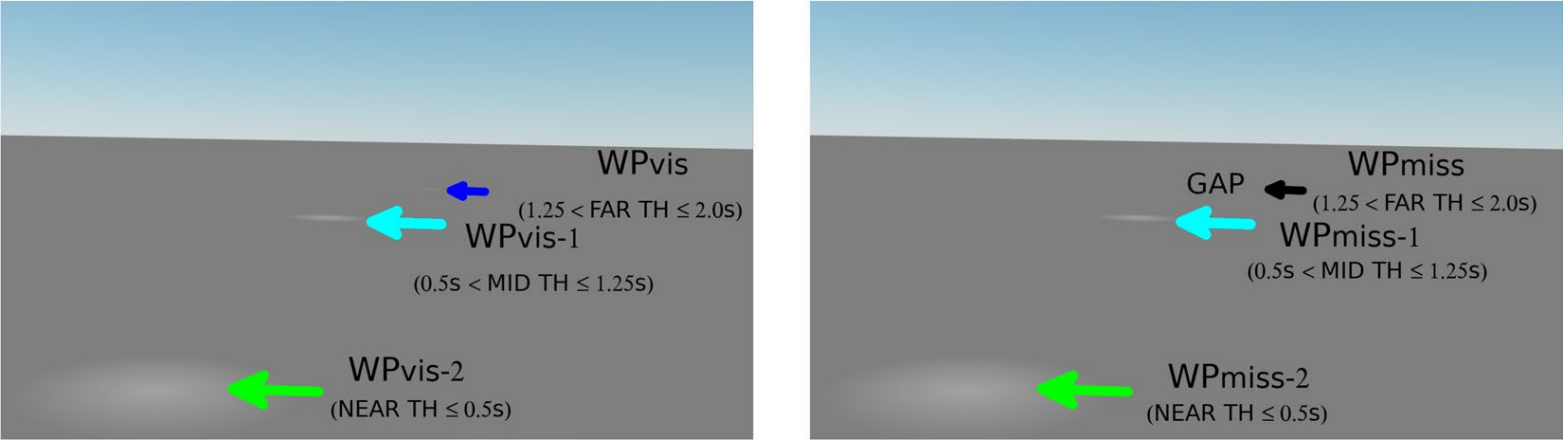
Waypoint presentation and naming convention. Every 0.75 s a new waypoint appears (WP_VIS_, left panel) at a TH 2 s, apart from the occasional Gap events (WP_MISS_, right panel). Thus, one, two or three waypoints could be visible at one time (the previously generated waypoint WP_VIS-1_/WP_MISS-1_ is at this point a TH = 1.25 s, and the waypoint WP_VIS-2_/WP_MISS-2_ before that is still visible at TH = 0.5 s). The labels FAR, MID, NEAR designate ranges of waypoint time headways: FAR: 2.0 > TH > 1.25 s; MID: 1.25 > TH > 0.5 s; NEAR TH < 0.5 s. The labels were not visible to the participant, and the actual displays were much larger than in this figure so WP_VIS_ was more obvious (see Movie 4 for a sample trial of the experiment).

The nearest waypoint disappeared from view (by passing below the virtual camera’s field of view) when it was approximately 3 m in front of the participant, and a new waypoint appeared every 0.75 s (Fig. 4). Thus, up to three waypoints could be visible at the same time (a new waypoint at TH = 2.0 s, a closer waypoint at TH = 1.25 s, and the closest waypoint at TH = 0.5 s, about to disappear from view at the bottom of the screen). Throughout the manuscript, waypoints at 2.0 > TH > 1.25 s are referred to as in the FAR range (at that moment in time), waypoints at 1.25 > TH > 0.5 s are in the MID range, and waypoints at TH < 0.5 s are in the NEAR range (borrowing the near/far criteria of visual field regions loosely from^42^ and^6^, although their models dealt with travel points, not waypoints).

Since the spatiotemporal qualities of the waypoints (in Experiment 2) were matched to the spontaneous waypoint tracking in Experiment 1, similar pursuit tracking of the waypoints was expected. The crucial question, however, was whether gaze would target the future path waypoint locations even when a visible target was *not* presented. Would saccades be directed toward a location where the (predictable) waypoint failed to appear or kept on the remaining visible waypoint? To investigate this, in 25% of the cases there was a gap event introduced between visible waypoints (Fig. 4): i.e. there was 1.5 s and twice the typical distance between waypoints (two gaps were never presented in succession). In effect, there was a ‘missing waypoint’ between two visible waypoints.

The idea was to probe whether drivers *predictively* shift their gaze to their future path without bottom-up visual cues (steering points specified by lane edges or moving ground texture). Specifically, if participants *predictively* track *waypoints* participants would be expected to perform saccades into the gap/missing waypoint, even when there was no visual stimulus present. This contrasts to *reactively* maintaining gaze on visible waypoints (which predicts pursuing the farthest visible waypoint for longer in a gap event, until the next waypoint after the gap appears). Fixating *travel points* – as per e.g. the popular Salvucci & Gray model^6^ and tangent point hypothesis^10^ – would not predict this pattern of smooth pursuits and saccades. Observing saccades directed to the missing waypoint would seriously undermine the plausibility of any purely bottom-up online control strategies – as there is no identifiable external stimuli present that would elicit the driver to shift their gaze to the future path – and suggests that top-down processing plays an important role in directing the drivers’ gaze in this task.

**Figure 5:**
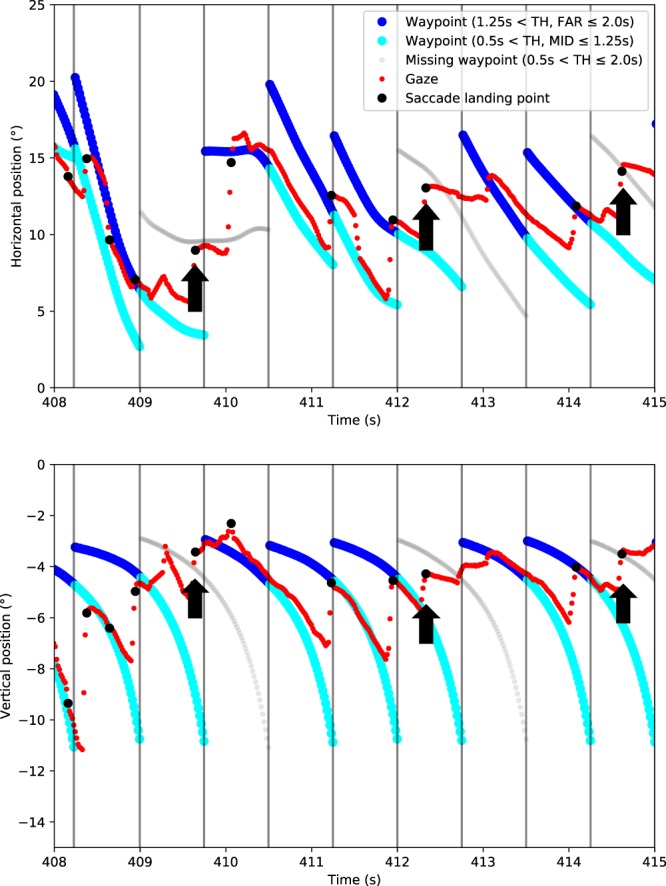
Gaze and waypoint time series. *Top*. Horizontal gaze position (red), and saccade landing points (black dots). Vertical lines indicate when a new waypoint (potentially) becomes visible, thick cyan/blue curves indicate the waypoints. Thin grey curves indicate missing waypoint locations. Blue indicates visible waypoints in the FAR range (1.25 s < TH ≤ 2.0 s), cyan indicates waypoints in the MID range (0.5 s < TH ≤ 1.25 s; note that as a new waypoint is created, the waypoint in the FAR range moves into the MID range). Continuation of waypoint position traces to the NEAR range omitted for clarity. Note that gaze very clearly tracks the waypoints with pursuit eye movements (but only in the FAR/MID range, switching then to the next waypoint with a saccade), and what appear to be anticipatory saccades into the gap/missing waypoint (black arrows), i.e. saccades landing where the waypoint would be. *Bottom*. Otherwise the same time series but with vertical positions.

Figure 5 shows a sample time series of a participant’s horizontal and vertical gaze position in relation to the positions of the different waypoints (including the ‘position’ of the missing waypoint – the location where a visible waypoint would have been if there had not been a gap).

**Figure 6:**
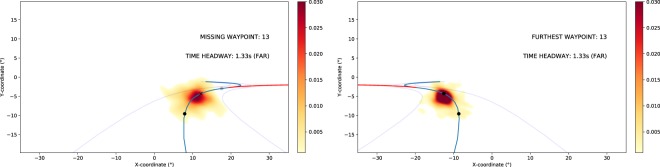
Sample frames from Movie 5 [left panel] and Movie 6 [right panel]. *Left panel*. The gaze density distribution in each of the different waypoint positions of the track (across all participants and trials), when there was a missing waypoint in the FAR range. Movie 5 shows how the gaze mass is centred on the missing waypoint location (x) between visible waypoints (black dots). This demonstrates anticipatory targeting of the predicted location of missing waypoints. In the video Index (1–16) in the upper right corner indicates missing waypoint location on the track. Each 1.5 s sequence begins at the time the (non-rendered) missing waypoint at the location marked with X would become visible in a non-gap event, i.e. 2 s before arriving at its position, and ends when the TH to the missing waypoint reaches the NEAR range (TH < 0.5 s). Grey dot: waypoint that is not yet visible, but will be visible after 0.75 s. X: missing waypoint location (gap). Black dot: visible waypoint. Blue line: Track centre. Red line: Track centre corresponding to a constant radius path, if there were no change in track curvature. *Right panel*. Movie 6 is otherwise comparable to Movie 5, but it displays the gaze density distribution in each of the different waypoint positions of the track (across all participants and trials) when there was a visible waypoint in the FAR range.

Videos of the gaze distribution densities in each separate waypoint location are available in the supplementary material (Movies 5 and 6), with separate videos for the visible waypoint sections of the road and the sections of the road with missing waypoints. Figure 6 show sample frames of these videos.

### Gaze frequency at different waypoint areas of interest

Figure 5 clearly shows an individual making saccades and fixating close to the invisible waypoint. To assess whether this trend was consistent across participants we needed a way of capturing fixation proximity to waypoints. The relative frequency of gaze (‘gaze catch’) in areas of interest (AOI) centred on putative visual targets is a standard way of investigating aggregate gaze distribution data.

**Figure 7:**
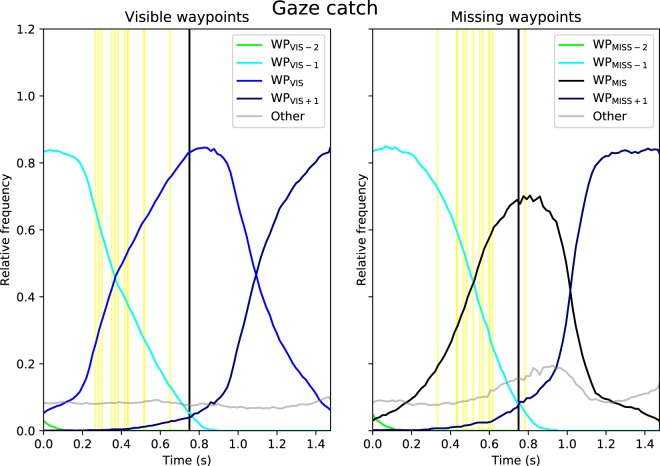
Relative frequency of gaze catch (with all data pooled) at different waypoint (WP) positions. *Left panel*. All WPs are visible. *Right panel*. All WPs are visible except for the missing waypoint WP_MISS_ (black line). The figure shows the 1.5 s period beginning (time = 0 s) from the moment that the visible WP_VIS_ appears on the screen [left panel] or when the missing WP_MISS_ would have appeared on the display if it were visible [right panel], and ending when the (visible or invisible) waypoint gets to TH = 0.5 s, i.e. enters the NEAR range. Black vertical lines indicate TH = 1.25 s when WP_VIS_ or WP_MISS_ moves from the FAR range to the MID range (and a new waypoint becomes visible in the FAR range). The yellow vertical lines indicate participant-wise crossover points where the mean gaze catch at the visible waypoint WP_VIS_ becomes higher than at the preceding WP_VIS-1_ [left panel] and similarly where the gaze catch at WP_MISS_ becomes higher than at the preceding visible waypoint WP_MISS-1_ [right panel]. Only data from road sections corresponding to constant curvature driving were included in the analysis (i.e. waypoints located where path curvature changes were excluded, see the Methods for details).

Left panel of Fig. 7 shows gaze catch % at different waypoint AOIs: a visible waypoint (WP_VIS_) in the FAR/MID range, the preceding waypoints WP_VIS-1_ (in the MID and NEAR range) and WP_VIS-2_ (in the NEAR range), and the future waypoint WP_VIS+1_ which becomes visible at time 0.75 s. The right panel shows a gap event with a missing waypoint WP_MISS_, and the (visible) waypoints WP_MISS-2_, WP_MISS-1_ and WP_MISS+1_. If gaze was >4° from any of these predefined locations it was classified as ‘other’.

When all waypoints are visible, participants shift gaze between waypoint WP_VIS-1_ and a newly visible FAR waypoint WP_VIS_. The crossover point when WP_VIS_ gaze catch becomes higher than WP_VIS-1_ is about 0.3–0.4 s after the appearance of the new waypoint. This pattern of tracking the current waypoint and moving to a new waypoint is fairly consistent across participants (Fig. 7, left panel).

Crucially, when the critical waypoint is invisible the AOI catch pattern remains similar to the visible waypoint events. Instead of continuing to fixate/pursue the preceding waypoint WP_MISS-1_ through the MID range, the missing waypoint WP_MISS_ AOI catches a substantial portion of the gaze distribution mass (although the crossover point is delayed by a hundred milliseconds or so; Fig. 7, right panel). Although in total participants look to WP_MISS_ less often (i.e. area under the black curve in Fig. 7, right panel) than WP_VIS_ (area under the central curve in Fig. 7, left panel), and a higher proportion of gaze is spent looking elsewhere (grey; Fig. 7; right panel), the pattern of switching waypoints is still robust. This is most clearly shown when the missing waypoint is at TH 1.25 s – the last moment before WP_MISS+1_ becomes visible. Despite there being no waypoint in the MID/FAR range to look at, at this moment the AOI catch at the WP_MISS_ is higher than the previous waypoint WP_MISS-1_ for all but one of the 12 participants (Binomial test, p = 0.006). The crossover point between WP_MISS_ and WP_MISS-1_ was later than the crossover point between WP_VIS-1_ and WP_VIS_ for all but one participant (Binomial test p = 0.006), with a mean difference of 0.12 s (SD = 0.08).

### Saccadic behaviour

The results presented so far show that there is an identifiable point at which participants’ gaze is on average closer to the missing waypoint WP_MISS_ than to the preceding WP_MISS-1_. However, this does not necessarily indicate that participants actively look at the missing waypoint. A reasonable alternative hypothesis could be that participants simply stopped pursuing the preceding waypoint and stared at the screen before the next waypoint in the FAR region appeared. As WP_MISS_ naturally moves closer (assuming they continue along the track) its location on the screen would get close to where the gaze is staring – even though WP_MISS_ was never actively targeted in the absence of a necessary bottom-up cue – and lead to spurious gaze catch at the WP_MISS_ AOI. There would not, however, be clear evidence of saccades towards WP_MISS_.

The final 0.25 s period when the missing waypoint is at the FAR range (time window 0.5–0.75 in Fig. 7) was used for investigating saccades landing in WP_MISS_ AOI. The data from the first 0.5 s were excluded to avoid spuriously assigning corrective saccades to the waypoint WP_MISS-1_ as targeting WP_MISS_. In other words, only saccades while the missing waypoint TH was between 1.25–1.5 s were analyzed. (With missing waypoints at positions 1,2,15 and 16 excluded due to their proximity to where the direction of the track curvature changes, see the Methods for details).

We examined to see if there was a significant decrease in the number of saccades when the waypoint in the FAR range was missing. The frequency of saccades in the last 0.25 s before the appearance of the next waypoint (i.e. WP_MISS+1_ or WP_VIS+1_) was smaller for WP_MISS_ compared to WP_VIS_ (t-test for per-participant differences, p < 0.001, Cohen’s d = 1.45), but there was nevertheless a substantial amount of saccades in the WP_MISS_ scenarios (even within the strict 0.25 s time window there were saccades in 29% of WP_MISS_ events, compared to 41% of WP_VIS_ events). In other words, though significantly less the participants were still saccading when there was no visible waypoint in the FAR range.

**Figure 8:**
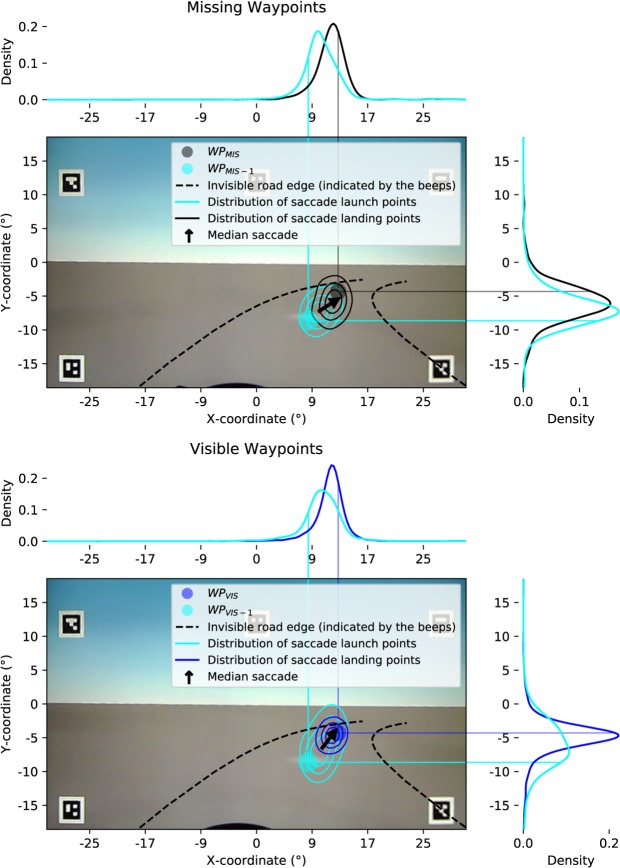
Saccade launch and landing points in the visual field for missing (top) and visible (bottom) waypoints. Top. Distribution of the saccade launch and landing points with regard to the missing waypoint WP_MISS_ in the last 0.25 s while it’s in the FAR range (during constant curvature driving, waypoints 3–14, see the Methods for details) Bottom. The same distribution but in regards to visible waypoints in the FAR region. Because the saccades were not always performed at the exact same time and the drivers were not always at the exact same position in relation to the track centre, the gaze coordinates were normalized along the vector between the waypoint WP_MISS-1_/WP_VIS-1_ in the MID range and the waypoint WP_MISS_/WP_VIS_ in the FAR range (so that the MID waypoint was at (0, 0) and the visible/missing FAR waypoint was at (0, 1)) and then reprojected back to the screen coordinates (left turning road sections mirrored). The density functions are Gaussian kernel density estimates. The median saccade was calculated as the vector between the median launch and landing points. The background image is a screen capture from the eye tracker’s forward camera.

The distribution of the saccade launch and landing points for the visible waypoints and for the missing waypoints in the FAR range can be seen in Fig. 8. Here, as in the case of AOI gaze catch (seee Fig. 7), saccades landed, on average, closer to WP_MISS_ than WP_MISS-1_ (t-test for per-participant differences, p = 0.001, Cohen’s d = 1.27). More to the point, the saccades tended to move towards WP_MISS_ rather than away from it (the landing points were closer to WP_MISS_ than the launch points were; t-test for per-participant differences, p < 0.001, Cohen's d = 1.33), with a mean shift of 1.8 degrees. This indicates active saccades in the direction of the missing waypoint WP_MISS_.

**Figure 9:**
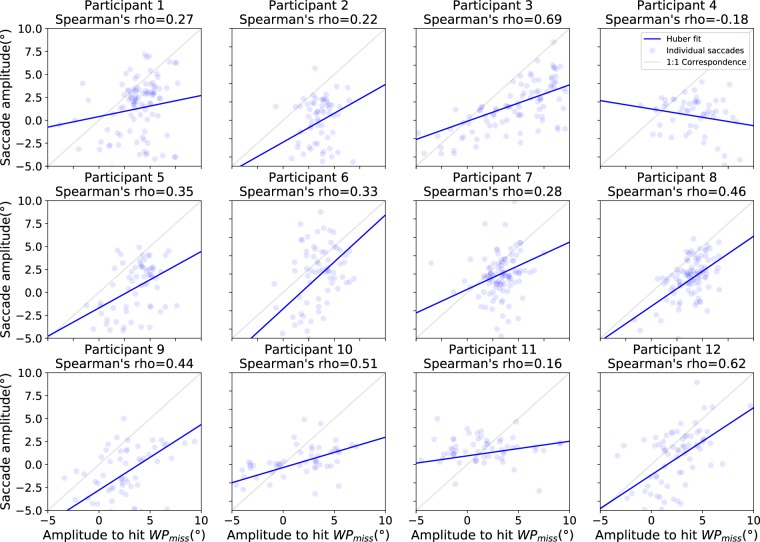
Participant-wise scatter plots between the horizontal amplitude of saccades and the horizontal amplitude that would be required for the saccades to hit WP_MISS_. The blue lines are the corresponding linear fits using the Huber loss function. These plots capture whether the saccades moved along the horizontal axis of the screen in the direction, and by the amount, that they should have done if they were to perfectly target the invisible waypoint horizontally. The median absolute horizontal amplitude to hit WP_MISS_ is around 3.6 degrees and saccade amplitude around 2.2 degrees.

There was a significant amount of variation in both the horizontal amplitudes of the saccades (SD = 3.5 degrees on the horizontal axis) and where WP_MISS_ was located on the screen (SD = 3.0 degrees on th ehorizontal axis). To examine if the variation in the saccade amplitudes could be accounted by the variation in the amplitudes that would be required to target WP_MISS_ from where the gaze was at the onset of the saccades (i.e. to see if the saccades where adaptive to the location of WP_MISS_ and/or where the saccade was launched from), we determined for each participant the Spearman’s correlation coefficient (see Fig. 9) between the horizontal saccade amplitudes (SACCADE_LANDx_ - SACCADE_LAUNCHx_) and the horizontal amplitudes between the saccade launch points and WP_MISS_ locations (WP_MISSx_ - SACCADE_LAUNCHx_). The amplitude between the saccade launch point and missing waypoint position tells us the (horizontal) amplitude that would be required for the saccade to hit the missing waypoint perfectly from where the saccade was launched from, while the (horizontal) saccade amplitude tells us what the actual amplitude was. By performing a Fisher’s z transformation on the correlation coefficients, we yield a (retransformed) mean correlation coefficient of 0.36 (with all but one participant having a positive correlation).

**Figure 10:**
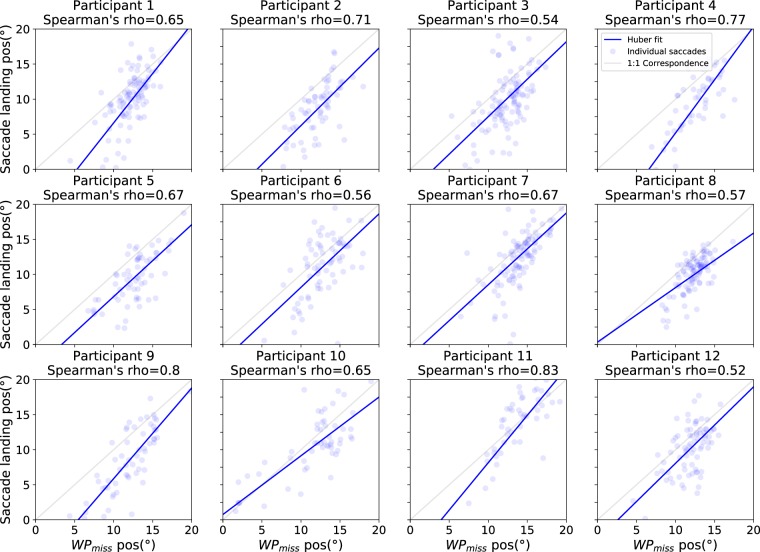
Participant-wise scatter plots between positions of WP_MISS_ and saccade landing points on the screen horizontal axis. The blue lines are the corresponding linear fits using the Huber loss function. The light grey lines indicate the 1:1 correspondence if the saccades perfectly target the invisible waypoint horizontally.

In addition, the horizontal position of saccade landing points was moderately correlated with the horizontal position of WP_MISS_ (see Fig. 10). The retransformed mean of Fisher’s z transformed Spearman’s correlations yields an average of 0.67. This indicates that the participants did not target any single point on the screen but rather directed their gaze to approximately where WP_MISS_ would have been if it had been visible.

## Discussion

We designed two experiments to investigate spontaneous gaze behaviours while steering a curving path, and in particular the nature of gaze control when local path information was withheld.

In Experiment 1 we quantitatively assessed spontaneous guiding fixations during an extended path following sequence, without artificial task instructions or road-edges that may have influenced gaze. Guiding “fixations” (that were clearly pursuit-like in nature) were used in a manner consistent with accounts of tracking a waypoint on the future path^33^. These waypoints were tracked for approximately 0.4 s as they approached (within a 1.5–3 s time window) before gaze switched onto the next waypoint.

In Experiment 2 we investigated how gaze was used to sample information when the future path was only specified by visible waypoints, and in particular how participants behaved when a waypoint was occasionally witheld. We observed that participants were able to steer along the path with only intermittent waypoints as input (without any visual information from path edge lines or ground flow, see Steering Performance in the Supplementary Analyses and Results for details on steering performance). Furthermore, we demonstrated that drivers actively shifted their gaze to (the vicinity of) the missing waypoint, even though there was always at least one alternative visible waypoint that they could have continued to pursue. There is no clear explanation for this phenomenon based upon purely bottom-up mechanisms of gaze control, as there were no visual cues to drive these gaze shifts.

A natural interpretation is that the participants anticipated the location of the missing waypoint (or their own future path) based on an internal model based on prior experience (i.e. memory of the preceding path). This result speaks to a long-running debate in the steering control literature: whether humans use internal models in guiding visual locomotion. Our results suggest that, at least as far as the active gaze strategy of visual guidance is concerned, they do.

It is worth considering whether there are alternative model-free interpretations that could account for these results. The eye movement patterns elicited in this task were fairly repetetive so a somewhat *ad hoc* explanation could be that the observed gaze patterns resulted from invoking a simple repeating motor program. Because of the fixed 0.75 s interval and the constant radius of the analyzed path segments, there was a somewhat consistent direction and distance between successive waypoints in the visual field. One simple rule could have been to generate saccades with equal magnitude and opposite direction to the previous pursuit. Another could have been to “default” to produce similar saccades as produced for preceding waypoints, if no new visible waypoints appeared. To test whether a simple model of producing saccades with a fixed frequency and constant amplitude might come close to explaining the observed behaviour, further analyses were performed on the relationship beween saccade amplitude generated and the amplitude that was needed to hit the missing waypoint (Fig. 9). Not only was there variation in saccade amplitude (suggesting a constant motor program was not adopted), there was a correlation between saccade amplitude and the amplitude required to target the missing waypoint.

Another possible simple control heuristic could have been for gaze to be targeted toward some typical point on the screen, one where the waypoints/path usually fell. This would have generated saccade amplitudes that varied depending on the saccade launch points. Further analysis (Fig. 10) suggests that this is also not a likely explanation for our findings. The position and rotation of the virtual vehicle actually caused variations in the screen location of missing waypoints. Furthermore the landing points of saccades on screen was not constant, and instead they were correlated with the actual location of the missing waypoint (Fig. 10).

The bottom line is that the spontaneous gaze strategies adopted shifted gaze from visually salient targets towards locations with no distinctive visual features, but where predictable and task-relevant visual information could be expected to appear in the near future. And these locations were not at fixed screen positions, nor was the amplitude of the saccades needed to reach these locations constant.

*Post hoc*, one may further postulate a purely oculomotor memory representation (a preprogrammed saccade sequence, that depends on some complex rules based on the preceding saccade history). Such a mechanism would not be dependent on a “world model” in spatial memory, but purely motor memory (e.g. stored efference copies). But this approach could not really be classified as purely online control, and begs the question as to why and how such heuristic strategies could arise in a model-free context. Unless the complexity of the saccade-generating-rule were constrained in some principled way, this control solution is not obviously more theoretically parsimonious than the internal-model account. (Spatial memory internal models *are* in a principled way constrained - e.g. by the geometrical layout of the road).

## Online vs. Model-Based Control

Humans display complex active gaze strategies when steering visually specified paths. In discussions of visually guided locomotion and action, there is often a distinction made between whether the mechanisms of control can be considered online or model-based. Zhao and Warren^25^ differentiate between strong-online control where control is driven by readily available stimuli, strong-model-based control where control is driven by an internal world model, and hybrid models where both model-based offline-control and direct online-control are applied (see also^9,20^). To put it simply, the question is whether control is directly based on continuous sensory stimuli, such as optic flow, or whether internal models that are updated on the basis of sensory stimuli are utilized to predict states of the world in the temporary absence of sensory stimuli.

The close coupling of gaze and steering control has encouraged steering models that translate optically specified stimulus information more or less directly to steering commands^5,6,10,33,40,42–44^. Such models adequately capture qualitative and quantitative characteristics of steering performance (at least during routine steering at moderate speeds). However, from the point of view of a mechanistic understanding of gaze-steering coupling, there are important gaps in this modelling framework.

First, they all deal with steering control *after* relevant perceptual information has been sampled from a particular location in the visual field. The crucial question of how, where and when information is sampled in the first place (the decision of where to (re)direct the eyes before new perceptual information is sampled) is not modelled explicitly; it is usually simply assumed, based on empirical observations, that the gaze is directed one of various possible steering points (for review see^8^). However, unless the steering point itself is specified by optical features (such as the tangent point described in^10^; or a designated target object to intercept, e.g.^45,46^ – the origin of this gaze/locomotor target information remains somewhat mysterious, unless one posits maintenance of an internal representation of the desired future path of some sort. This is a challenge for the majority of models that use online control processes to steer relative to a “desired” *reference trajectory* that is needed to compute feedback (steering error).

Second, the models usually rely on measures that are defined in the observer’s *locomotor heading* frame of reference – such as the visual direction of a steering point used for guidance – while physiologically the visual information is always obtained in the retinal frame of reference^5^. A full account of visuomotor control of gaze and locomotion requires specification of this mapping process between retinal data and steering action, which is likely quite elaborate in naturalistic tasks, such as driving, where typically multiple gaze targets are sampled in rapid succession^13^. How the mapping between the input and output frames of reference is achieved is fundamentally different in model-based versus online control. An online mechanism would continuously implement the full mapping between stimuli (optical information) and actions (motor commands), without auxiliary mediating representations (e.g.^10,45,47^). In model-based control, sensory information is integrated into an internal model, which among other things must continuously maintain up-to-date knowledge of the relationship between the frames of reference (cf.^48–50^, for discussion in the context of visually guided locomotion, see^51^).

The argument for model-based control is supported by occlusion studies where car drivers are able to maintain control for several seconds without visual feedback^52–56^. Similar observations have been made in other domains: human subjects are able to walk blind to predesignated targets^57–59^, catch thrown balls in the dark^60–62^, and point at objects during brief visual occlusion^63,64^. These observations would appear to be rather strong arguments in support of locomotor control based on internal models (cf.^65,66^; see also^67^).

Also, given that the involvement of prediction is commonly assumed in studies of visual tracking based on anticipated target motion^68^ (see also^69^), and saccades and pursuit generation is accounted for by predictive models^70,71^, it may seem surprising that the role of internal models and predictive processing is so highly contested in the field of visual steering control theory. One reason is that given the many potential cues in natural tasks, it is not straightforward to show that performance could not plausibly be explained by some online control heuristic or another.

## Conclusions

In Experiment 1 we saw that with a visually specified path and rich optic flow, gaze appears to spontaneously track waypoints as the observer moves toward them, then seeking out a new target location further up the road. In Experiment 2 we investigated these gaze control processes further by only specifying the path using waypoints, and then occasionally withholding this waypoint information. We saw that gaze sought out the waypoint locations with anticipatory saccades, even when the visual stimulus was not presented.

This rules out simple bottom-up visual transitions or salience as explanations of gaze control in this active steering task. It demonstrates that the mechanisms responsible for targeting waypoints can produce behaviour that is difficult to explain solely with online control, or simple stored motor programs, but is consistent with predictive internal models integrating information over time and across fixations. It should be noted that given the fairly simple geometric layout and task dynamics these need not be complex, geometrically detailed and metrically accurate full world models - rather simple representations of the future path should be sufficient. We should also emphasise that the results do not show that steering control is *not* informed by directly available information (such as optic flow or target visual direction). However, if actions were *entirely* guided by such immediate visual information, without any internal model of the desired trajectory, why would gaze apparently seek out the missing waypoints on the future path? More to the point, if no predictions were involved, *how* would it do so? What information specifies the saccade target position? More extensive manipulations of the nature of the (un)predictability of the waypoints in future experiments should be able to clarify these underlying representations and constrain the development of explicit theories of integrated gaze-steering coordination based on predictive processing.

## Methods

### Driving simulator

The simulator setup consisted of a distance-adjustable gaming chair (Playseat Evolution Alcantara, Playseats B.V., The Netherlands), a steering wheel (In Experiment 1: Logitech G25, Logitech, Fremont, CA. In Experiment 2: Logitech G920 Driving Force, Logitech, Fremont, CA) and a 55 LG 55UF85 monitor. The experiments were run on an HP ENVY Phoenix 860-081no (Intel Core i7-6700K CPU, NVIDIA GeForce GTX 980 TI GPU) desktop computer with Linux Debian as the operating system. The gaming chair was placed at 85 cm from the monitor, creating approximately a 70° horizontal field of view of the monitor to match the virtual field of view of the simulator. The height of the seat was not adjustable and thus eye height varied slightly between participants, but the participants’ eye height was approximately in line with the centre of the monitor.

The simulation was run at 1920 × 1080 pixel resolution in a 60 Hz frame rate. Virtual camera eye height was approximately at 1.5 m from the ground. Engine noise was played through the screen’s loudspeakers.

The software for the driving simulator was developed in-house and is available as open source (code for EXP1: https://github.com/prinkkala/webtrajsim, code for EXP2: https://github.com/samtuhka/webtrajsim/tree/fixswitchsim).

### Eye tracker

In both experiments, the participants’ eye movements were recorded with a binocular head-mounted Pupil Labs eye tracker (Pupil Labs UG haftungsbeschränkt, Berlin, Germany). The open-source Pupil Capture software was used to record and calibrate the eye tracker, specifically, a custom fork (https://github.com/samtuhka/pupil) of the software was used with some edits from the base version, such as custom placed calibration marker locations and slightly adjusted pupil detection parameters.

Eye image data was recorded binocularly at 30 Hz, at a resolution of 640 × 480 for the eye image cameras. The eye tracker was calibrated with 22 calibration markers that were displayed one by one on the monitor screen. The calibration was performed by determining a polynomial fit by the least-squares method from the centre positions of the pupil to the detected marker positions. In experiment 1, the calibration accuracy was measured at the end of the six trials with 22 calibration markers placed at both the centre and edges of the screen. The mean calibration accuracy was 1.16 degrees with a standard deviation of 0.67 degrees. For one participant the calibration accuracy could not be measured as the world camera of the eye tracker appears to have been significantly moved directly after the six trials. In experiment 2, the calibration of the eye tracker was measured (mean accuracy: 1.27 degrees, SD: 0.93 degrees) and adjusted after each trial with six calibration markers placed within ±15 degrees from the centre of the screen.

In addition, five 5 × 5 square optical markers were placed on the edges of the screen (see Fig. 1) to determine in each video frame a projective transformation from the eye tracker’s forward-camera to the monitor coordinates by the correspondence of the edges of the optical markers in the video frame to their known screen coordinates. In effect, the optical markers were used to compensate head movements and allow us to calculate the position of the gaze on the monitor screen.

### Gaze signal analysis

For each recorded gaze point, a confidence rating was derived from the ratio between the length of the detected pupil edge and the circumference of the fitted pupil ellipse. All data below a confidence rating of 0.6 was omitted from the analysis. The omitted data wasn’t analyzed in depth but was largely isolated to individual frames or occasionally a period of a few seconds at most.

**Figure 11:**
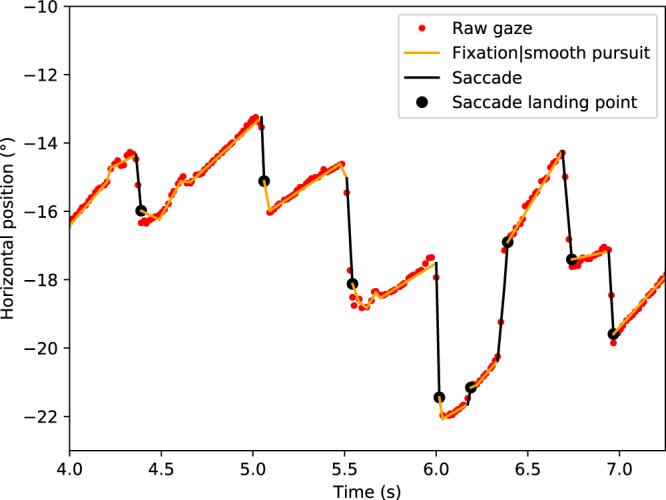
Sample time series of a participant’s horizontal gaze positions and saccade identification in Experiment 1. Red dots depict gaze position in screen coordinates, black lines are saccades identified by the segmentation algorithm, black dots indicate saccade landing points, and red lines non-saccadic events (pursuit, fixations).

The gaze position signal, which had been projected to the monitor coordinates, was de-noised and classified into individual saccades and smooth-pursuit segments with the naive segmented linear regression (NSLR) algorithm^72^. NSLR approximates a maximum likelihood linear segmentation from the data points (i.e. data is modelled as linear segments between initiation and termination points of successive segments). The associated Hidden Markov Model classifier (NSLR-HMM)^72^ was used to identify different segments into saccades, fixations, smooth pursuits and post-saccadic oscillations. The saccade identification appeared to be the most reliable, whereas the other classifications often seemed hard to differentiate from one another, so for our purposes we used the saccade landing and launch points (which respectively are also the end and start points of the prior and successive segments) to approximate the start and end points of guiding fixations. For an example in the resulting signal see Fig. 11. In the AOI catch analysis, we used all of the (calibrated) gaze points from the eye tracker.

### Ethics

The experiment was conducted at the TRUlab at the University of Helsinki. The study was approved by the ethical review board of the University of Helsinki and followed the Helsinki declaration and guidelines of the Finnish committee for research ethics (www.tenk.fi). Upon arrival, the participants were debriefed as to the purpose of the study, and they signed an informed consent form for the publication and use of the collected data for scientific purposes.

### Experiment 1 participants

In Experiment 1, a convenience sample of 15 participants (9F, 6M, mean age = 30, SD = 7, range = 20–48 yrs) was recruited through University of Helsinki mailing lists. All of the participants had at least 30,000 km of driving experience and reported normal or corrected vision. The participants were compensated with two activity vouchers worth €10 in total for participation.

### Experiment 1 stimuli and design

**Figure 12:**
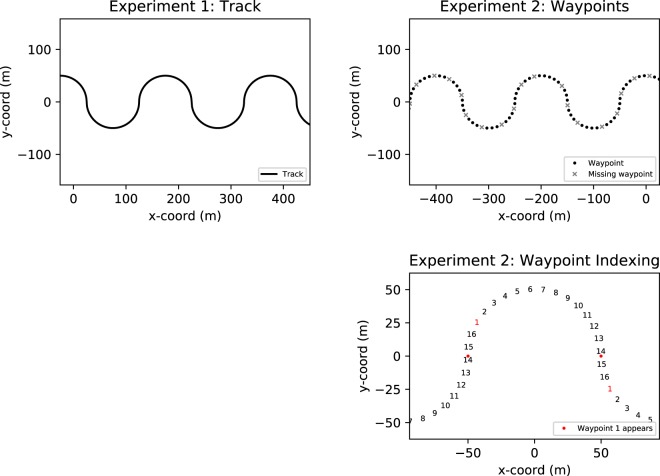
The virtual track layout. *Top left*. Bird’s eye view of a portion of the path in Experiment 1. Each semicircle had a radius of 50 m and arc of 180°. *Top right*. Waypoint locations in Experiment 2. The selection of the missing waypoints (i.e. which waypoints would not be rendered, marked with X) differed in each trial (the panel shows one possible configuration). Bottom right. The indexing of the different waypoint locations in a bend. During a single turn, there 16 waypoint locations altogether (at 0.75 s intervals). The indexing begins with waypoint 1 which appears (unless there is a gap) at a 2 s time headway when the driver crosses the point where the sign of road curvature changes (red dot).

The track consisted of 50 m radius half-circles that alternated to right and left (Fig. 12). The path width was approximately 3.5 m in total with 0.60 m of shading on both sides. The participants drove the track through six trials with three different speeds: 40 km/h, 53 km/h, and 66 km/h (two trials of each). A single trial lasted for approximately one minute. The speed of the virtual vehicle was automatically kept constant at the chosen speed and the participants controlled the vehicle only with the steering wheel. The trials were ordered from the lowest speed to the highest, always starting with the lowest speed and ended with the highest. The participants were instructed to ‘drive along the track’ and stay on the path. No gaze instructions or instructions to keep to the middle of the path were given.

The road and ground textures were designed to give realistic-looking visual flow and were piloted to reduce aliasing at the natural fixation distance (which can create a steady-state appearance visual artefact). The ground and road textures were generated with power-law noise (beta = 2.0)^73^. This produces textures with a frequency-amplitude relationship typically observed in natural images^74^.

### Experiment 2 participants

A sample of 12 participants (10F, 2M, mean age = 27, SD = 5, range = 21–36 yrs) was recruited through University of Helsinki mailing lists. All of the participants reported having at least 20,000 km of driving experience and a normal or corrected vision. The participants were compensated with two activity vouchers worth 10 in total for participation.

### Experiment 2 stimuli and design

Like in Experiment 1, the participants drove through a track consisting of 50 m radius, 180° arc half-circles alternating to left and right. Unlike in Experiment 1, a single trial took approximately two minutes and the track had ten turns/half-circles (see Fig. 12). Also, unlike in Experiment 1, the ground texture had been removed and replaced with solid grey colour to keep the visual flow of the ground at zero. The speed of the virtual car was automatically kept constant at approximately 47 km/h (corresponding to 15°/s yaw rate on the 50 m radius half-circles). The track width was 3.5 m in total. If the participant strayed off from the track (i.e. veered more than 1.75 m from the centre of the track), the simulator started playing a loud beeping sound as a warning (after the practice phase this was the only cue of the track width). If the participant drove more than 10 m off the track, the trial was restarted.

In the *practice phase* the participants drove through the track twice with a visible road present. The road texture of Experiment 1 had been replaced with a solid white colour (see Fig. 1, centre panel). The purpose of the practice phase was to get the participants comfortable with the dynamics of the car and gain an understanding of the track.

In the *test phase* the road was hidden from sight and the only visual cues of the track were waypoints. The waypoints were 0.7 m radius circles on the ground with a grey-white texture that faded linearly to blend in with the grey ground texture toward the edges (see Fig. 1, right panel). The upcoming waypoints were not immediately visible, but rather each waypoint appeared when it was from the driver’s position at a 2 s time headway along the track – assuming the driver would keep a constant 15°/s yaw rate along the circular track from that point.

The waypoints were equally spaced-out (at 0.75 TH intervals, corresponding to the waypoint appearance frequency), with occasional larger gaps (when only near-visual-information would be available for a small period of time) of 1.5 s. To be precise, the intervals were determined by travelled distance on the track, not time per se (i.e. the intervals were approximately 9.8 m or 19.6 m).

When a new waypoint appeared its opacity linearly increased from full transparency to maximum opacity in 0.25 s. The purpose of this fade-in was to minimize the pop-out effect of a new waypoint appearing in the scene. The new waypoint began to appear 0.75 s after the previous one but in 25% of the cases, there was a gap. There were always 4 such gaps in a single half-circle with never more than 1 gap in succession.

In other words, in a single half-circle, there always were 16 (180°/15°/s/0.75 s = 16) potential waypoint locations. In effect, there were 16 waypoints with 12 visible waypoints and 4 missing waypoints in one such turn. The different waypoint possible locations were identified with by index numbers 1–16 (see Fig. 12). The gaps/missing waypoints could occur in any of the locations, but because the waypoints were the only visual cues of the path in order to prevent the participant veering off the track, two successive locations could not both have a gap. In addition, there was always a minimum of two visible waypoints between missing waypoints.

In the practice phase, the participants were instructed to drive on the road. They were informed in the beginning that later on in the test phase of the experiment the road would be hidden from sight and they would have to drive on the basis of only occasional ‘road markings’. Beyond that, the only instruction was to keep on the lane and complete the track. The participants drove through the track twice.

In the test phase, the participants were told that they would be driving on the same track as in the practice phase, but now the road had been hidden out of sight and its only visual indicators would the be occasional waypoints (or ‘road markings’ as they were called in the instructions). The participants were explicitly told that the track, with the exception that sometimes the first turn was to the left and sometimes to the right, was the same shape as in the practice phase. They were also informed that the locations of the road markings might change in different trials, but no further details on the underlying logic of the waypoint placement or the gaps were provided. The participants drove through the track 8 times in the test phase. Whether the first turn was to left or right was randomized, as well as the locations of the gaps.

### Experiment 2 data analysis

The data from sections corresponding to the waypoint locations 3–14 (see Fig. 12) was pooled together in the analysis to visible waypoint sections and to missing waypoint sections depending on whether there had been a gap or not. The road sections close to the turning points (waypoint locations: 1, 2, 15 and 16) were not included the analysis for the sake of simplicity – the different constant curvature sections can reasonably be assumed to be more comparable to each other than the curve exit/entry sections.

In the analysis of saccadic behaviour, the gaze signal was similarly segmented as in Experiment 1. For the AOI analysis, we determined in monitor projected coordinates for each gaze position the nearest waypoint location of interest on the screen. The gaze position was then assigned to the AOI of the waypoint location. These were the current screen positions of the waypoints in the NEAR, MID and FAR range – or in case of the ‘missing waypoints’, the location where a waypoint would normally have been, and the location of the next upcoming waypoint (TH > 2.0 s). If gaze was >4° from any of these predefined locations, the gaze location was classified as *‘Other’*.

The AOI catch during the 0.75 s periods between waypoint appearances is mostly at either the waypoint in the MID range or at the waypoint/missing waypoint in the FAR range. The catch at the waypoint in the NEAR range drops quickly to almost zero, and there is slight rise in the catch of the upcoming waypoint (TH > 2.0 s) at the end, but the waypoints (even the missing waypoints) in the MID and FAR range are by far the most dominant. Because the gaze catch at the other locations was relatively small, when examining where people look during the gap events, we only analyzed the AOI catch differences between the waypoint in the MID range and the missing waypoint in the FAR range.

The purpose of the AOI catch analysis was to examine how much of the participants’ gaze was directed to the missing waypoint instead of the waypoint in MID range in order to test our hypothesis that the participants would shift their gaze to the gap of continuing to pursuit/fixate the middle waypoint. Due to reaction times and the fact that it normally took 0.25 s until the far waypoint was at maximum visibility, we did not assume the participants would do so *immediately*. Instead, a cutoff of 0.5 s (from the point when a waypoint would appear if there was no gap) was used as the point in time from which on we examined whether the drivers’ gaze was on average found at or near the waypoint in the MID range or at the missing waypoint. For comparison, at 0.5 s the catch frequency at the waypoint in the MID range had dropped down to 25% (averaged across all participants) when the waypoint in the FAR range was visible. We applied this cutoff of 0.5 s to the missing waypoints (i.e. we compared the sum of AOI catch at the two different locations during the 0.5–0.75 s interval).

## Supplementary information

Supplementary Analyses and Results
