# Supplementary material for: Humans Use Predictive Gaze Strategies to Target Waypoints for Steering
Source: Sci Rep. 2019 Jun 6;9:8344. doi: 10.1038/s41598-019-44723-0 (PMC6554351; doi:10.1038/s41598-019-44723-0)
Supplement: Supplementary file 1 — Supplementary Analyses and Results [file 41598_2019_44723_MOESM1_ESM.pdf]

Humans Use Predictive Gaze Strategies to Target Waypoints During Steering

Authors: Samuel Tuhkanen[1, 2], Jami Pekkanen[1, 2] , Paavo Rinkkala[1, 2] , Callum Mole[3], Richard M. Wilkie[3] & Otto Lappi[1, 2]

[1] Cognitive Science, Department of Digital Humanities & Helsinki Centre for Digital Humanities (Heldig), University of Helsinki, FINLAND
[2] TRUlab, University of Helsinki, FINLAND
[3] School of Psychology, University of Leeds

Supplementary Analyses and Results

Saccade Time Headways in Experiment 1

We made the observation that the time headway of the guiding fixations in Experiment 1 consistently decreased as the speed increased (see **supplementary table ST1** for individual participant results). The limited field of vision of the simulator (and the fact that the participants were not able to rotate the virtual camera other than by rotating the car) is a likely concern here. For example, at the maximum speed of 66 km/h, the maximum visible time headway can be as low as 3 seconds (the maximum visibility varies on the basis of the location and rotation of the vehicle in respect to the path). While the observed time headways were even lower (mean saccade landing point TH: 1.76 s) it seems at least intuitively plausible that the drivers would avoid directing their gaze close to the edge of the screen even if in a more unconstrained setup they would look further. The fact that the trials were ordered from lowest speed to the highest speed is also a possible factor – if the participants increased familiarity with the track has an effect on where they look it would naturally coincide with the increase in speed. In order to test this further, future experiments have to be done with randomized speed profile in virtual reality with free-head movements that correspond to the movement of the virtual camera or in the field, alternatively larger curve radii could elicit higher visibility in terms of time headway.

One other possible explanation involves the increased difficulty of lane keeping with higher speeds (indicated by the higher variation in travel path deviation and increased time spent outside the path, see Steering Performance, below, for details). Land and Horwood [1] demonstrated in a simulator setting that when drivers are only presented with 'far' visual information about the road, i.e. everything else except a small segment of the screen was occluded, the driver's lane keeping ability significantly deteriorated. Vice versa the loss of 'far' information negatively affected the driver's ability to estimate the road curvatures. Similarly, in the steering model of Salvucci and Gray [2] where steering is guided by a near and far travel point, the near-point information is more integral to lane keeping whereas far-point information is used for greater overall stability. It may be that as the difficulty of lane keeping increases with speed (note that the speed was automatically kept constant, the participants were not able to control it), more guiding fixations fall on the near-vicinity of the driver whereas in easier conditions the drivers are able to distribute more attention to the 'far zone'.

It should also be noted that while the time headways decreased with speed, rather than staying constant or possibly even decreasing, the distance at which saccade landing points fell did increase with speed, just not as much as would be predicted if the time headway stayed constant (see **Supplementary Figure S1**).

Supplementary Table ST1. Median saccade launch & landing point time headways and median duration between saccades (in seconds) for each participant in each speed condition in **Experiment 1**.

Participant	Launch point time headway (40 km/h)	Landing point time headway (40 km/h)	Duration between saccades (40 km/h)	Launch point time headway (53 km/h)	Landing point time headway (53 km/h)	Duration between saccades (53 km/h)	Launch point time headway (66 km/h)	Landing point time headway (66 km/h)	Duration between saccades (66 km/h)
1	2.29	2.6	0.32	1.71	2.02	0.36	1.38	1.64	0.45
2	2.69	3.08	0.62	1.93	2.25	0.54	1.44	1.87	0.48
3	2.26	2.77	0.33	2.1	2.4	0.39	1.73	1.95	0.56
4	2.08	2.38	0.4	1.71	1.93	0.34	1.55	1.83	0.31
5	2.89	2.92	0.26	2.06	2.17	0.29	1.89	2.0	0.27
6	2.77	2.87	0.41	2.06	2.28	0.43	1.61	1.85	0.58
7	2.34	2.48	0.41	2.07	2.35	0.32	1.73	1.91	0.28
8	2.16	2.37	0.31	1.74	1.95	0.33	1.44	1.69	0.33
9	1.74	2.05	0.33	1.55	1.7	0.47	1.48	1.54	0.36
10	2.36	2.55	0.46	1.8	2.05	0.37	1.55	1.72	0.36
11	2.22	2.43	0.2	1.58	1.78	0.21	1.42	1.59	0.16
12	1.99	2.31	0.41	1.52	1.78	0.32	1.47	1.7	0.35
13	2.85	3.11	0.34	2.31	2.51	0.32	1.75	1.88	0.3
14	2.22	2.4	0.35	1.82	2.11	0.38	1.27	1.55	0.44
15	2.58	2.82	0.55	2.04	2.24	0.52	1.58	1.73	0.47
Grand Mean	2.36	2.61	0.38	1.87	2.10	0.37	1.55	1.76	0.38

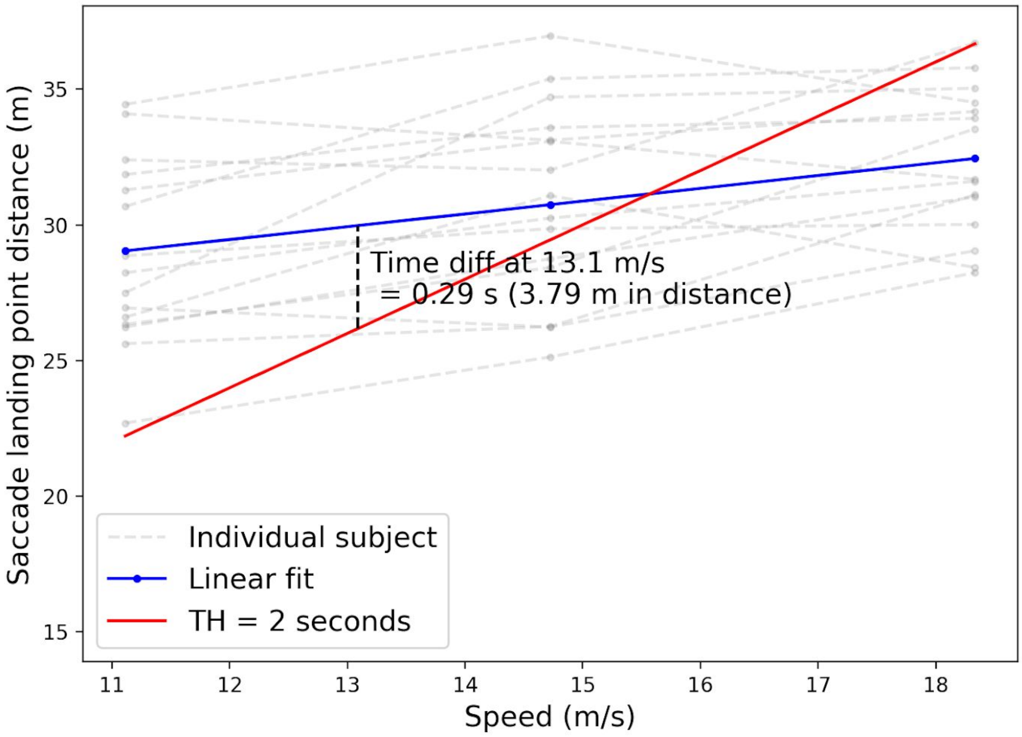

Supplementary Figure S1. Saccade landing point distance (curvilinear distance in regard to the trajectory) as a function of speed in **Experiment 1**. Each dotted grey line indicates the median saccade landing point distance of an individual participant in the three speed conditions. The blue line is the linear fit ($r = 0.40$, $p = 0.006$) across all participants. The red line indicates constant time headway of 2 seconds. As the speed increased, all participants looked further ahead, but not by enough to maintain a constant time margin. The time difference between the linear fit and the 2 seconds time headway (the distance at which the waypoints appeared in **Experiment 2**) is 0.29 seconds at 13.1 m/s (the speed used in **Experiment 2,** yaw rate of 15°/s). The 2s TH is thus conservative and not “too far” for a typical participant.

Steering Performance

While the gaze behaviour of the drivers was our main interest, we also chose to examine how well the drivers were able to steer in Experiment 2 in comparison to Experiment 1 in terms of both deviation from the track and steering smoothness. The participants (of Experiment 2) mainly stayed on the track or quickly returned if the warning sound started to play, but there were 2 separate occasions on which the participant was unable to return to the track (specifically they drove over 10 meters off the track) after the end of a turn. Overall however the participants’ ability to stay on the lane was comparable to the two higher speed conditions of Experiment 1 (see **Supplementary Table ST2**).

Supplementary Table ST2: Mean distance from lane the centre and time spent out of the lane. Positive numbers refer to oversteering and negative to understeering.

Experiment & condition	Mean distance from lane centre	Time out of the lane (i.e. distance from lane centre > 1.75 m)
EX 1 (40 km/h)	0.60 m	0.1%
EX 1 (53 km/h)	0.71 m	1.9%
EX 1 (66 km/h)	0.66 m	2.3%
EX 2 (47 km/h)	-0.25 m	1.8%

In terms of deviation from the centre of the path/road, there is a small bias which is in opposite directions in the two experiments (**Supplementary Figures S2** and **S3**). In **Experiment 1** the participants appear to steer closer to the inner edge of the road whereas in **Experiment 2** the participants appear to steer closer to the outer edge of the road. Note that in contrast to many steering studies, we did *not* instruct the participants explicitly to steer at the centre of the road (the waypoints in Exp 2 were in the middle, so arguably a central lane position was implied) in either experiment. Thus, constant bias should not be construed as steering error.

The variability (SD) in lane position was about 25-30cm (**Supplementary Figure S4**). In particular, using only the waypoints in Experiment 2 does not create more lane position variability than the textured fully visible road in Experiment 1, except at the reversal points where the sign of the path curvature changes. When the target information is not available in these transitional locations (when the gap is in the turn position following the curvature zero crossing). The participants cannot then reliably predict that the path will make an S i.e. the turn direction will reverse, and they can get momentarily “lost” (evidenced by peaks in SD, **Figure S4**).

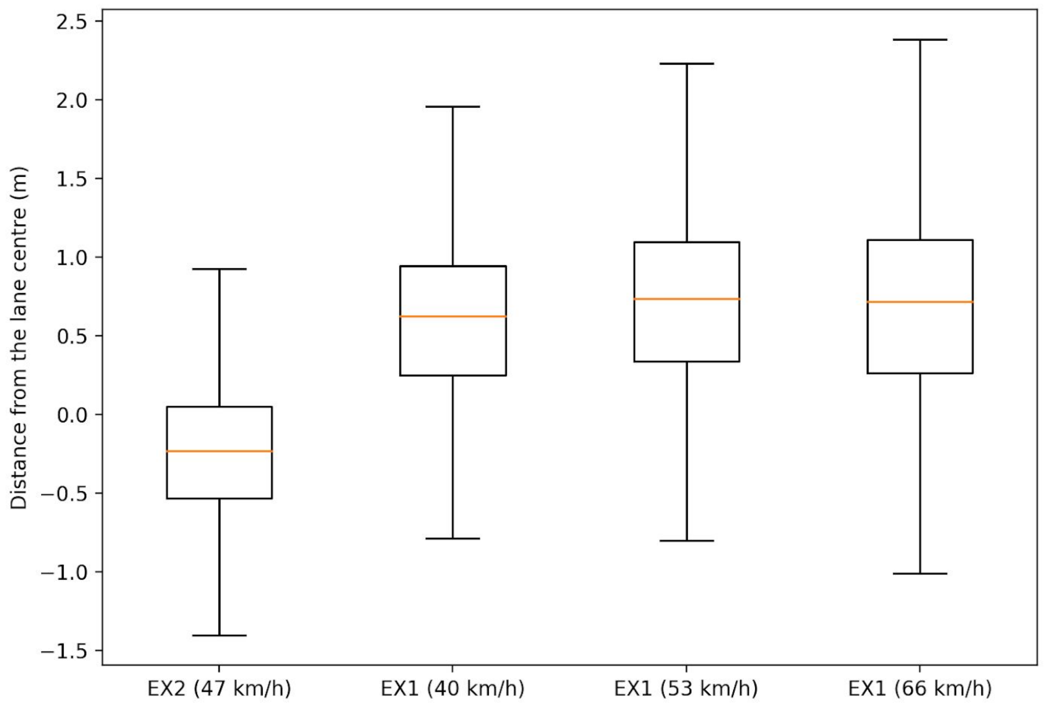

Supplementary Figure S2. Boxplot of the distance from the path centre (i.e. the centre of the lane). EX2 = **Experiment 2** and EX1 are the three speed conditions of **Experiment 1**. The whiskers extend to 1.5*IQR from the lower and upper quartile. Positive numbers mean toward the inner edge (oversteering) and negative toward the outer edge (understeering).

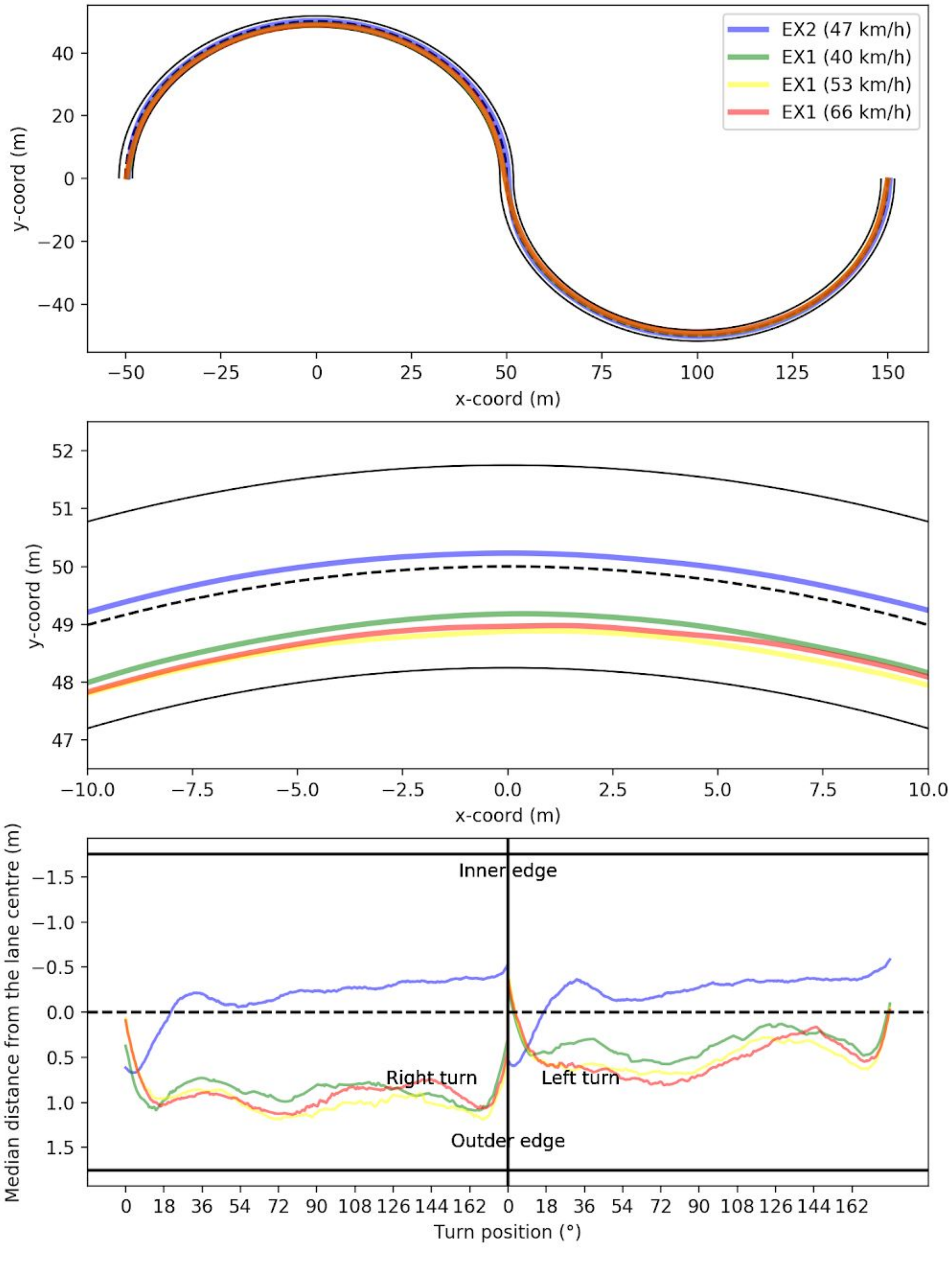
EX2 (47 km/h)
EX1 (40 km/h)
EX1 (53 km/h)
EX1 (66 km/h)
y-coord (m)
x-coord (m)
y-coord (m)
x-coord (m)
Inner edge
Right turn
Left turn
Outder edge
Median distance from the lane centre (m)
Turn position (°)

(previous page) **Supplementary Figure S3.** *Top*. The median travel path over all participants in **Experiment 1 (**three speed conditions, **green, yellow** and **red)** and **Experiment 2** test phase (**blue)**. The "median path" was determined by calculating the median x, y coordinates in each turn position (i.e. for every data point the closest point on the path was determined and the x,y medians of each of those points were determined). The solid black lines indicate where the road texture has fully faded out in **Experiment 1** and the threshold where the alarm 'beep' is played in **Experiment 2** and (i.e. the effective road edges, although there is no painted edge line), the dotted line indicates the (invisible) lane centre**.** *Middle.* Close-up of the above. *Bottom.* Median distance from the lane centre over all participants in **Experiment 1 (green, yellow** and **red)** and **Experiment 2 (blue)**. Turn position in the semicircular bend runs from 0 to 180 degrees.

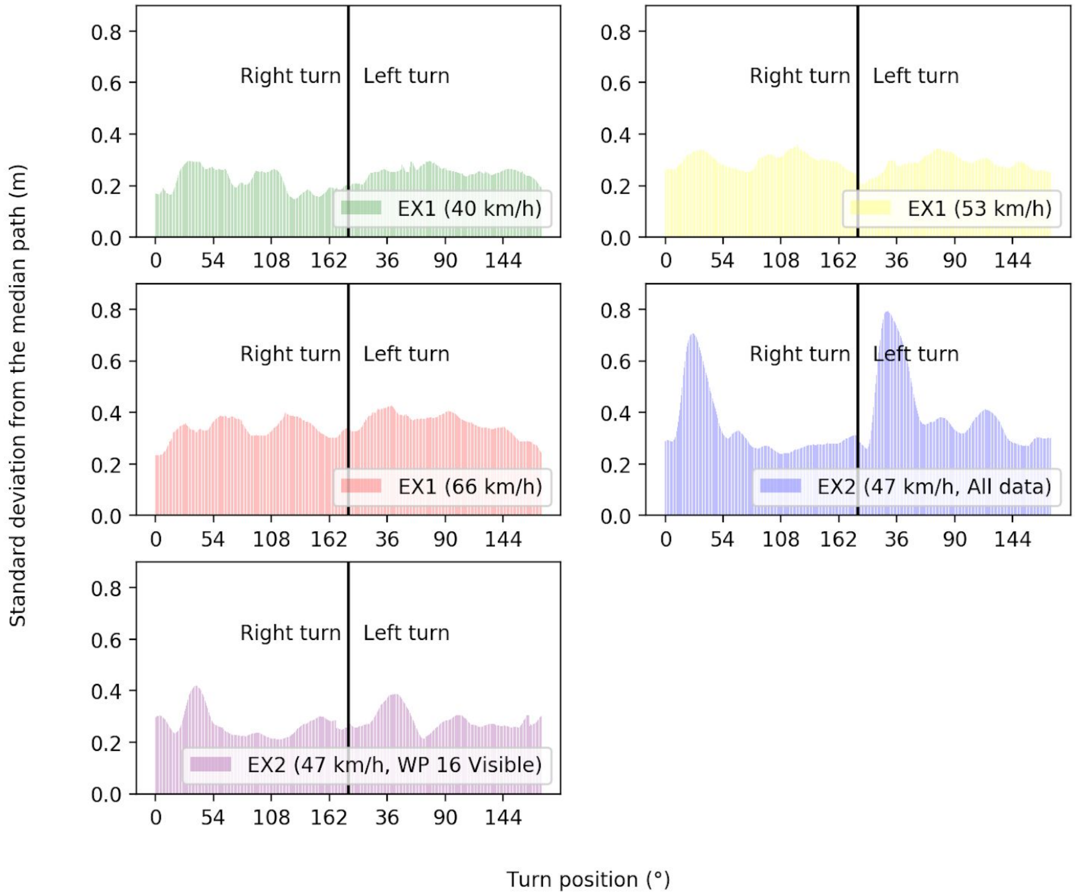

Supplementary Figure S4. Standard deviation from the median path as a function of turn position. **Experiment 1 (EX1** three speed conditions, **green, yellow and red)** and **Experiment 2** test phase with all data included **(EX2 blue)** and with turns with a missing waypoint 16 removed **(EX2 purple)**. In **Experiment 2** there is significantly more deviation near the beginning of a turn. Otherwise, the deviation from the median path appears to be comparable in both experiments, even though the travel paths themselves are different. Furthermore, most of the deviation at the beginning of the turns appears to be caused by the missing waypoint 16.

Because different steering wheels were used in Experiment 1 and Experiment 2, rather than examining steering acceleration directly we chose yaw acceleration as our measure of steering smoothness. Even though the participants in Experiment 2 were able to stay on the track, for the most part, the yaw accelerations are significantly higher in Experiment 2 (mean of participant means: $6.8°/s^2$) than in the two lower speed conditions of Experiment 1 (means: $2.2°/s^2$, $3.5°/s^2$, $6.2°/s^2$ for each of the respective speed conditions) even when only the constant curvature sections were analyzed (sections corresponding to waypoints 3–14 in Experiment 2).

Videos:

Movie 1
Movie 2
Movie 3
Movie 4
Movie 5
Movie 6

REFERENCES

[1] Land, M., & Horwood, J. (1995). Which parts of the road guide steering? Nature, 377(6547), 339-340.

[2] Salvucci, D. D., & Gray, R. (2004). A two-point visual control model of steering. Perception, 33(10), 1233-1248.
